# The antifungal effect induced by itraconazole in *Candida parapsilosis* largely depends on the oxidative stress generated at the mitochondria

**DOI:** 10.1007/s00294-023-01269-z

**Published:** 2023-04-29

**Authors:** Mª Luz Muñoz-Megías, Ruth Sánchez-Fresneda, Francisco Solano, Sergi Maicas, María Martínez-Esparza, Juan-Carlos Argüelles

**Affiliations:** 1 Facultad de Biología, Área de Microbiología, Universidad de Murcia, 30100 Murcia, Spain; 2 Departamento de Bioquímica, Biología Molecular B & Inmunología, Facultad de Medicina, Campus de Ciencias de La Salud, Universidad de Murcia, 30120 Murcia, Spain; 3 Departamento de Microbiología & Ecología, Facultad de Biología, Universitat de València, Burjassot, 46100 Valencia, Spain

**Keywords:** ROS, Rotenone, Dinitrophenol, Mitochondria, Trehalase, *Candida parapsilosis*

## Abstract

In *Candida parapsilosis*, homozygous disruption of the two genes encoding trehalase activity increased the susceptibility to Itraconazole compared with the isogenic parental strain. The fungicidal effect of this azole can largely be counteracted by preincubating growing cells with rotenone and the protonophore 2,4-Dinitrophenol. In turn, measurement of endogenous reactive oxygen species formation by flow cytometry confirmed that Itraconazole clearly induced an internal oxidative stress, which can be significantly abolished in rotenone-exposed cells. Analysis of the antioxidant enzymatic activities of catalase and superoxide dismutase pointed to a moderate decrease of catalase in trehalase-deficient mutant cells compared to the wild type, with an additional increase upon addition of rotenone. These enzymatic changes were imperceptible in the case of superoxide dismutase. Alternative assays with Voriconazole led to a similar profile in the results regarding cell growth and antioxidant activities. Collectively, our data suggest that the antifungal action of Itraconazole on *C. parapsilosis* is dependent on a functional mitochondrial activity. They also suggest that the central metabolic pathways in pathogenic fungi should be considered as preferential antifungal targets in new research.

## Introduction

The amazing spread of mycotic infections that affect the immnucompromised population (i.e., aging people, neonates, AIDS patients and those subjected to invasive surgery, prolonged treatments with antibiotics or suffering chronic diseases, etc.) and the rising identification in both community and hospitality epidemic outbreaks of fungal species traditionally considered as innocuous (Pfaller and Diekema 2010; Pfaller et al. 2014; Brown et al. 2012; Kainz et al. 2020) conveys additional complications for clinical antifungal chemotherapy, already limited by the scarce availability of true fungicidal compounds and the growing isolation of strains which display resistance to the conventional antimycotics (Kontoyiannis Lewis 2002, Denning and Bromeley 2015, Campoy and Adrio 2017).

The taxonomical complex *Candida parapsilosis* may be considered a significant example of this worrying scenario. The clinical incidence of this emergent yeast has increased in the last years and represents the second or third most common opportunistic fungal pathogen isolated worldwide (Trofa et al. 2008; Toth et al. 2019). As regards the antifungal treatments for *C. parapsilosis*, some specific pathobiological features must be borne in mind. For example, this yeast does not form true hyphae and is able to colonize the skin and produce stable biofilms on plastic-made devices, with a high incidence in neonates (Holland et al. 2014; Pal et al. 2021). As a general rule, although susceptibility testing is recommended for all clinical isolates, azoles appear to be useful, despite some reports of resistance whereas echinocandins have higher MICs values and display lower in vitro toxic activity compared to other *Candida* species (Cantón et al. 2011; Zhang et al. 2015). In turn, the new formulations of amphotericin B overcome the hepatic and renal toxicity caused by the classical formulations of this polyene and can be applied in adults (Saliba and Dupont 2008).

As a complementary strategy, the central nutritional pathways in pathogenic fungi have recently been proposed as preferential therapeutic candidates for new investigations into antifungals (Prieto et al. 2014; Wijnants et al. 2020; 2022). Within this area, the enzymatic routes involved in trehalose metabolism have received great attention since this non-reducing disaccharide is present in bacteria, fungi, plants and invertebrates, but is surprisingly absent from vertebrates, which, in turn, contain two trehalase isoforms responsible for the hydrolysis of trehalose ingested in the diet (Tournu et al. 2013; Perfect et al. 2017, Argüelles 2017; Thamagong et al. 2017). Furthermore, the genes encoding trehalose biosynthesizing enzymes (trehalose synthase and trehalose phosphatase) are factors of virulence and the corresponding null mutants display a phenotype of highly susceptibility to oxidative and saline stress exposure, also reported in *C. albicans* and budding yeasts (Pedreño et al. 2002; Van Dijck et al. 2002; Garre and Matallana 2009).

In our previous studies on trehalose metabolism in *C. parapsilosis*, we disrupted the two single genes encoding trehalase activity (*ATC1/NTC1*) in this yeast, and demonstrated their specific role in virulence (Sánchez-Fresneda et al. 2015). Thus, the homozygous *atc1Δ/ntc1Δ* null mutant, besides its inability to mobilize trehalose, displays a pleiotropic phenotype that includes the impairment of cell growth, resistance to nutrient starvation and oxidative stress, together with a reduction of virulence and the capacity to form biofilms (Sánchez-Fresneda et al. 2015). A recent analysis of azole susceptibility showed that *atc1Δ/ntc1Δ* cells were more sensitive to itraconazole (ITC) than the parental strain (Sánchez-Fresneda et al. 2022). We report here that the ITC-induced fungicidal effect is dependent on a functional mitochondrial activity.

## Materials and methods

### Strains and growth conditions

The *C. parapsilosis* strains used in this study have been reported elsewhere (Sánchez-Fresneda et al. 2015). Liquid cultures were grown at 37°C by shaking in YPD medium consisting of 2% peptone, 1% yeast extract and 2% glucose. Solid media contained 2% agar. The time-course growth in liquid medium was measured by monitoring cell density at 600 nm (OD_600_) or by direct cell counting in a TC-20 cell counter (BioRad). Cell viability was determined in samples diluted appropriately with sterile water b plating in triplicate on solid YPD after incubation for 1–2 days at 37°C. Between 30 and 300 colonies were counted per plate. Survival rates were normalized to control samples (100% viability). Colony growth in solid medium was tested by spotting 5 μl from the respective tenfold dilutions onto YPD agar. The plates were then incubated at 30°C and scored after 24 or 48 h.

### Preparation of cell-free extracts

After exposure to the indicated treatments, samples from YPD liquid cultures were harvested, washed and resuspended at known cell densities (10–15 mg/ml, wet weight) in the extraction buffer, 100 mM 4-morpholine-ethanesulfonic acid (MES) pH 6.0, containing 5 mM cysteine and 0.1 mM phenyl methyl sulphonyl fluoride (PMSF). Cellular suspensions were transferred into small pre-cooled tubes (1.0 cm-diameter) with 1.5 g Ballotini glass beads (0.45 mm diameter). The cells were broken by vigorously vibrating the tubes in a vortex mixer and rapidly cooled in ice. The crude extract was then centrifuged at 10,000 ×g for 5 min and the pellet was resuspended in the same buffer at the initial density. For antioxidant assays, the supernatant fraction obtained was filtered through Sephadex G-25 NAP columns (Amersham Pharmacia Biotech AB) previously equilibrated with 50 mM K-phosphate buffer, pH 7.8, to remove low-molecular-weight compounds that could interfere with the measurements.

### Enzymatic assays

Catalase activity was determined at 240 nm by monitoring the removal of H_2_O_2_ following the protocol described elsewhere (González-Parraga et al. 2011). Measurements of superoxide dismutase (SOD) were carried out spectrophotochemically by the ferricytochrome C method using xanthine/xanthine oxidase as the source of superoxide anions (O_2_^.−^) (González-Parraga et al. 2011). Protein was estimated using the Lowry method (1953) with bovine serum albumin as standard. Data of enzymatic activity were normalized in relation to a control measurement (100%).

### ROS determination

Intracellular ROS formation by flow cytometry with dihydrofluorescein diacetate (DHF), was measured following the procedure described in Sangalli-Leite et al. (2011) with the additional modifications indicated elsewhere (Sánchez-Fresneda et al. 2015). Fluorescence intensity was determined using the EPICS XLMCL4 cytometer (Beckman Coulter) equipped with an argon ion laser with an excitation power of 15 mW at 488 nm. Forward scatter (FSC) and side scatter (SSC) were analyzed on linear scales, while the analyses of green (FL1) fluorescence intensity were made on logarithmic scale. Analysis gates were set around debris and intact cells, on an FSC *vs* SSC dot plot. Fluorescence histograms corresponding to 5000 cells were generated using the gated data. Data acquisition and analysis were performed using WINMDI software (available from http://facs.scripps.edu).

## Results

### The antifungal effect of itraconazole on *C. parapsilosis* is counteracted by the presence of rotenone and DNP

**Fig. 1:**
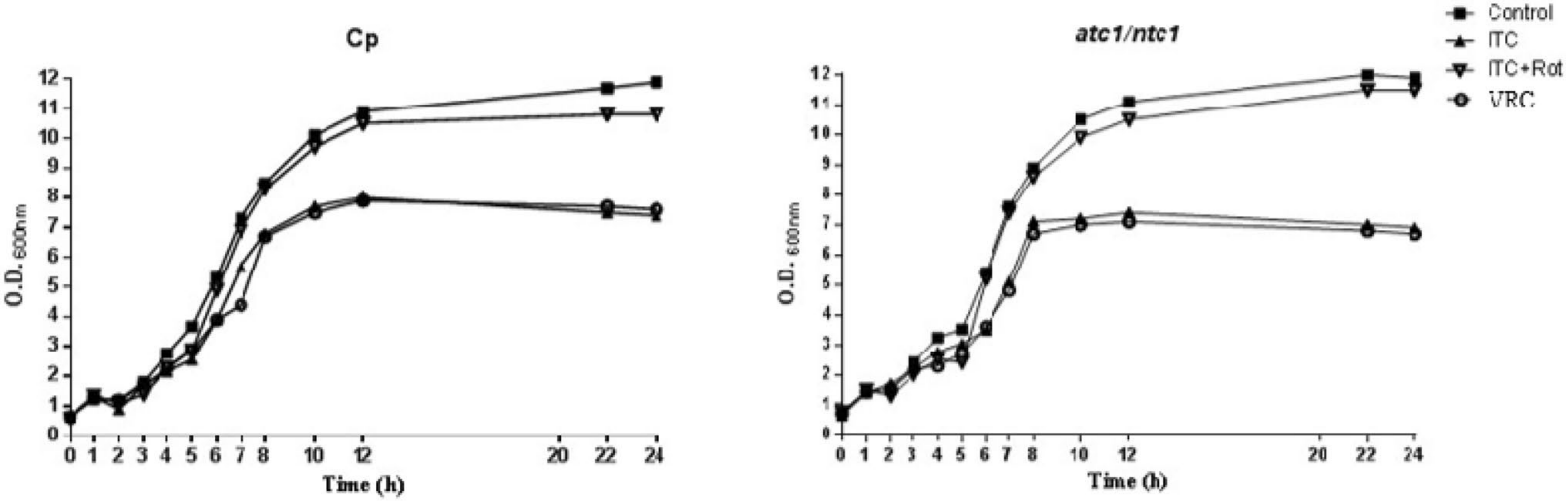
Effect of homozygous disruption of the two genes encoding trehalase activity in *C. parapsilosis* on cell susceptibility to Itraconazole (ITC) and Voriconazole (VRC). A pre-inoculum of both the parental strain of *C. parapsilosis* (Cp) and the trehalase-deficient null mutant *atc1Δ/ntc1Δ* were cultured overnight in YPD medium, diluted to an OD_600 nm_ of 0.3 in the same medium and further incubated at 37°C. Cellular growth was measured turbidimetrically by recording the changes in OD_600 nm_ during 24 h. Symbols: (■) Control, (▲) ITC (0.3 µg/ml), (○) VRC (0.3 µg/ml), (▼) (ITC + rotenone)

Neutral and acid trehalase activities in *Candida parapsilosis* are coded by two distinct genes, called *NTC1* and *ATC1* respectively, which are non-essential genes (Sánchez-Fresneda et al. 2015). Therefore, a double *atc1Δ/ntc1Δ* null mutant obtained by homozygous deletion of both genes is perfectly viable, although *atc1Δ/ntc1Δ* glucose-grown cultures displayed a weak phenotype of cell death after prolonged incubation in stationary phase (5 days onwards) (Sánchez-Fresneda et al. 2015). However, for the purpose of the ITC-induced fungicidal action presented in this study, only short-term incubations were carried out. As plotted in Fig. 1, the kinetics of cell growth in YPD medium at 37°C for one day measured by turbidimetry, were virtually indistinguishable between a *C. parapsilosis* parental strain (Cp) and its isogenic null mutant *atc1Δ/ntc1Δ*. Furthermore, this result also supports that an operative trehalase activity is unnecessary during the active growth cycle on glucose as carbon source (Fig. 1).

It has previously shown that treatment with Itraconazole (ITC) induced a significant toxic action on YPD-grown exponential cells of *C. parapsilosis*, recorded both as a decrease in the percentage of cell viability and the macroscopic formation of colonies (Sánchez-Fresneda et al. 2022). Indeed, the addition of 0.3 µg/ml ITC (equivalent 2 × MIC) also caused a prominent time-course reduction of the optical density (Fig. 1). Alternative inclusion of Voriconazole (0.3 µg/ml), another recently commercialized azole, gave rise to a similar phenotype (Fig. 1), suggesting that several azoles share a similar pattern of toxicity against *C. parapsilosis*. Although no experiments were followed beyond 24 h here, exposed cells were able to resume active growth after that time, since these compounds are essentially fungistatic (Sánchez-Fresneda et al. 2022).

**Fig. 2:**
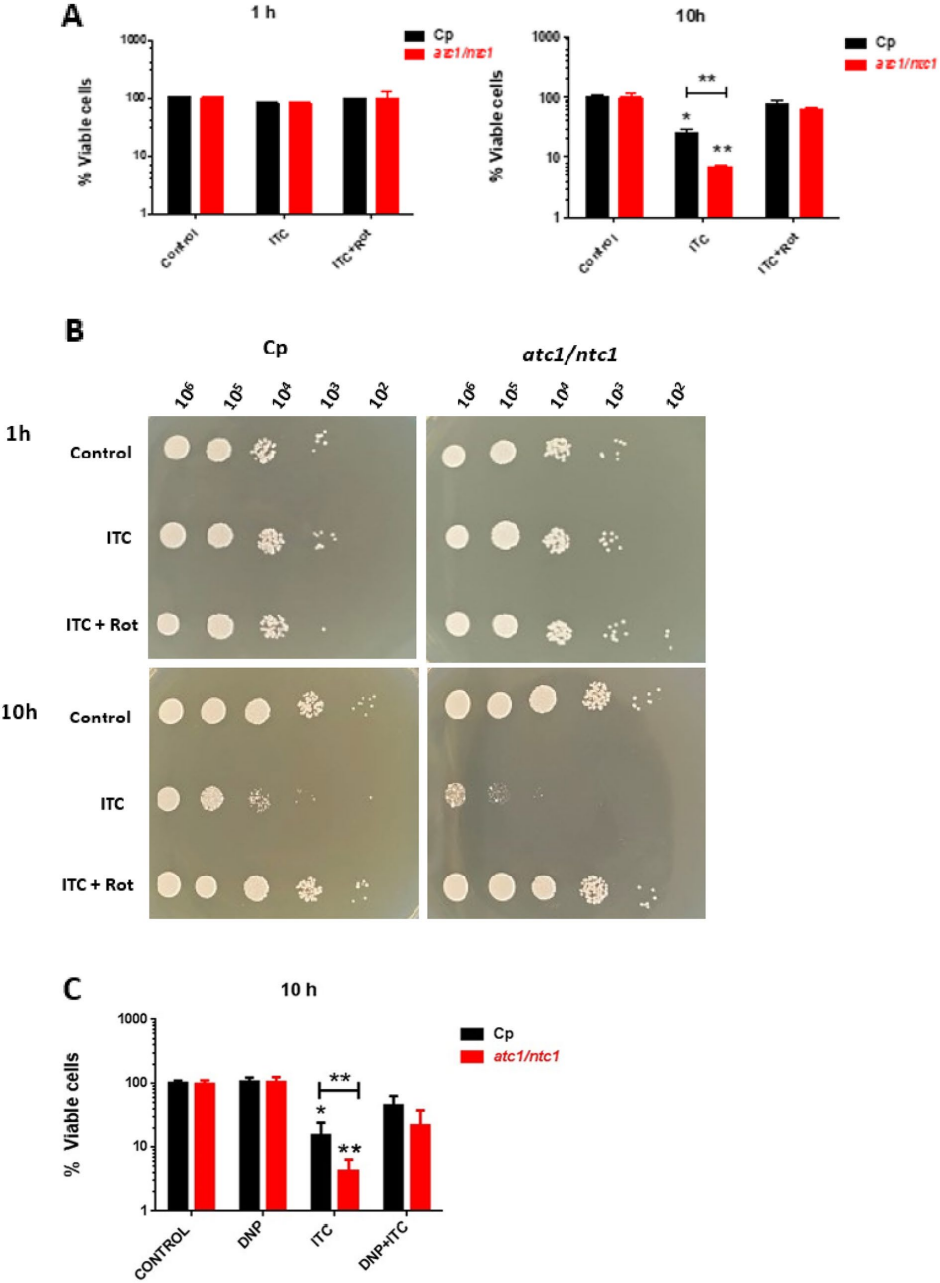
Protective action of rotenone and DNP on *C. parapsilosis* cell viabilitiy against the antifungal toxicity induced by addition of Itraconazole (ITC). YPD-grown exponential cells of the two cell types under study, were preincubated with either rotenone (0.156 mM) or DNP (0.5 mM) for 1 h at 37°C, and immediately treated with ITC (0.3 µg/ml) for 1 h or 10 h **A** and **B** or only 10 h **C**. Identical samples were then harvested, diluted appropriately, and spread on YPD plates. The percentage of cell survival was determined by CFU counting. The data shown are the mean ± SD of three independent experiments. *P < 0.05 and **P < 0.01 represent statistically significant differences with respect to an untreated control according to Mann–Whitney *U* test. **B** Tenfold cell suspensions were spotted on YPD plates (5 µl), which were incubated at 37°C and scored after 48 h

Apart from the well-established inhibition of ergosterol biosynthesis, consistent evidence points to the generation of inner oxidative stress as a contributory factor to the antimycotic action exerted by some azoles against relevant pathogenic fungi (Belenky et al. 2013; Shekova et al. 2017), as has previously been demonstrated with polyenes (Mesa-Arango et al. 2014). Therefore, we evaluated the hypothetical involvement of mitochondrial activity in the *C. parapsilosis* cell damage caused by exposure to Itraconazole, measured in terms of cell survival. For this purpose, an equivalent number of active cells from the two strains under study were pre-treated with rotenone (0.156 mM) for 1 h at 37°C. This subtoxic concentration is able to effectively inhibit the mitochondrial Complex I without any impairment of cell viability (Fig. 1; Mesa-Arango et al. 2014; Guirao-Abad et al. 2020). Then, 0.3 µg/ml ITC was added and the cultures were further incubated for 1 h or 10 h. As can be seen at Fig. 2 (A and B), the fungicidal activity of this azole was virtually absent after 1 h of treatment. However, longer incubation (10 h) led to Itraconazole having a strong toxic effect of on both *C. parapsilosis* strains, which could be counteracted by the presence of rotenone (Fig. 2A and B). The reduction in cell viability was also in accordance with the recorded turbidimetric measurements in the absence of rotenone (See Figs. 1 and 2).

Alternative experiments were carried out using the protonophore 2,4-Dinitrophenol (DNP). Notably, this inhibitor of oxidative phosphorylation induces resistance to Fluconazole in *Saccharomyces cerevisiae* (Kontoyiannis 2000). For comparative purposes, these results are presented in Fig. 2C, but only an assay after 10 h of exposure was processed, since ITC has no effect after 1 h (Figs. 2A and B). As can be seen, 0.5 mM DNP did not affect the percentage of cell viability whether in parental or in *atc1Δ/ntc1Δ* cells. However, preincubation with 0.5 mM DNP for 1 h partially counteracted the ITC-induced fungicidal action (Fig. 2C), although to a minor extent to that recorded with rotenone (Fig. 2). Furthermore, the *atc1Δ/ntc1Δ* null mutant always displayed higher susceptibility to ITC respect to the parental strain (Fig. 2; Sánchez-Fresneda et al. 2022). Hence, these results suggest that the antifungal effect triggered by ITC on *C. parapsilosis* is, at least in part, dependent on a functional mitochondrial activity.

### The Itraconazole effect takes place simultaneously with intracellular ROS formation

To corroborate this hypothesis, the level of intracellular ROS formation was measured simultaneously, since indirect data suggest that ROS generation takes place upon azole addition in *C. albicans* and *Aspergillus sp*. (Belenky et al. 2013; Shekova et al. 2017). In eukaryotic organisms, mitochondria are the main generators of endogenous ROS, which are formed as by-products of the electron respiratory chain. To check whether this mechanism is operative in the two cell types of *C. parapsilosis* following ITC exposure, the production of intracellular ROS was determined by flow cytometry using the highly specific staining with DHF (see Methods, Sangalli-Leite et al. 2011).

**Fig. 3:**
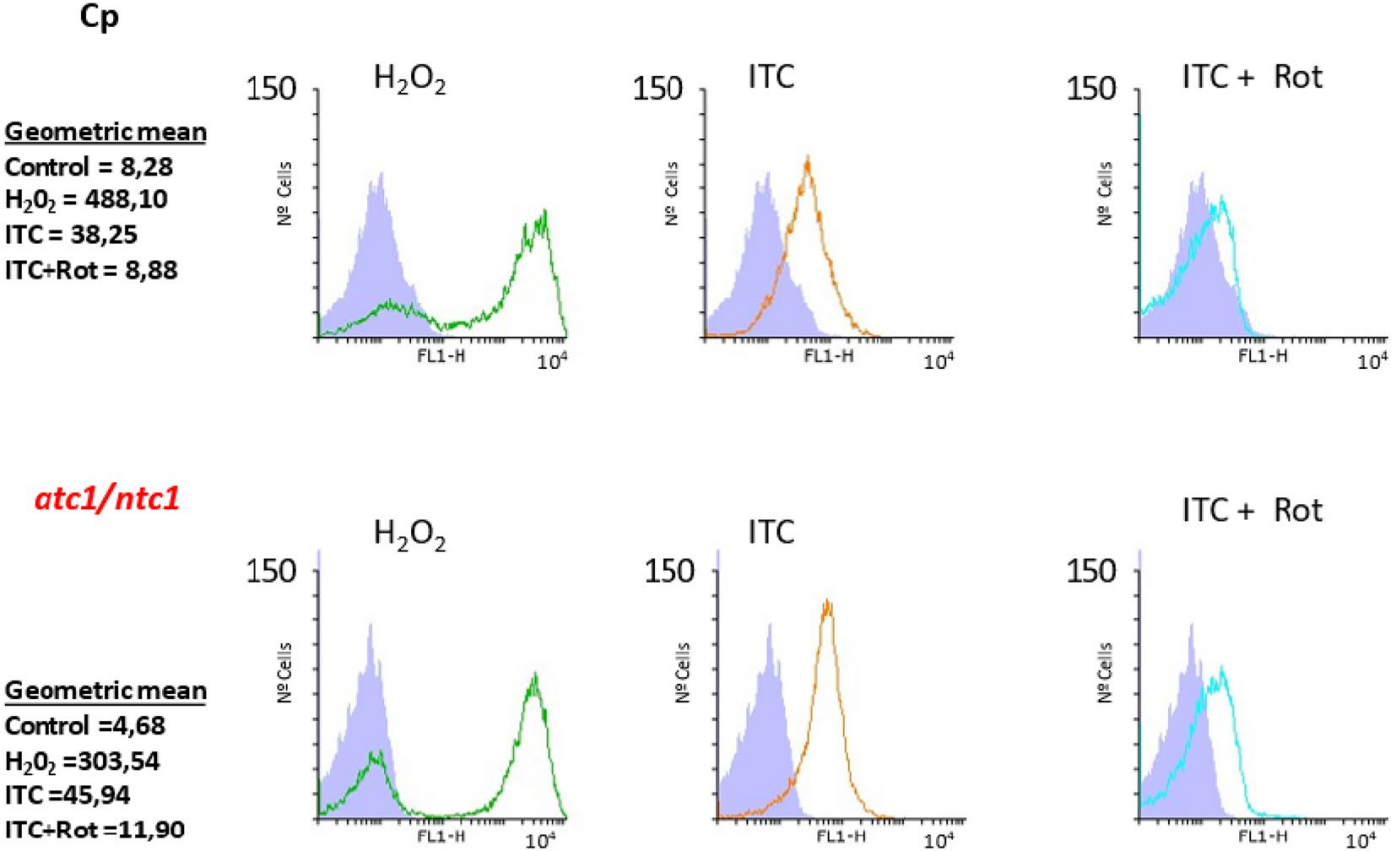
Level of intracellular ROS production after treatment with Itraconazol and protective role of rotenone in *C. parapsilosis*. Equivalent aliquots of YPD-grown exponential cultures of the two strains were incubated for 1 h in the absence or presence of rotenone (0.156 mM) at 37°C, followed by 1 h of treatment with ITC (0.3 µg/ml). ROS were quantified by flow cytometry using DHF in Cp (upper row histograms) and *atc1Δ*/*ntc1Δ* (lower row histograms) strains. The histograms display the cell number with respect to the green fluorescence intensity (FL1). A positive marker for acute oxidative stress (50 mM H_2_O_2_) was also introduced

In these experiments, a positive oxidative stress control (H_2_O_2_ 50 mM) was run in parallel. The results from a representative assay are shown in Fig. 3. As it has been previously reported (Sánchez-Fresneda et al. 2022) the *atc1Δ/ntc1Δ* mutant produced a lower basal level of endogenous ROS (Fig. 3). However, the fraction of cells able to generate intracellular ROS after ITC addition was higher in this trehalase-deficient mutant compared with its isogenic parental strain (Fig. 3; central panel). Notably, preincubation with rotenone led to a significant reduction in the capacity to produce endogenous ROS, which was rather similar in both strains, although the inhibitory action of rotenone seemed to be lower in control Cp cells (Fig. 3). This result might be due to the greater ITC-induced ROS production in *atc1Δ/ntc1Δ* cells (Fig. 3). Hence, we preliminary conclude that ITC can induce a significant oxidative stress through intramitochondrial ROS formation in *C. parapsilosis*.

### Antioxidant enzymatic activities in response to Itraconazole addition

**Fig. 4:**
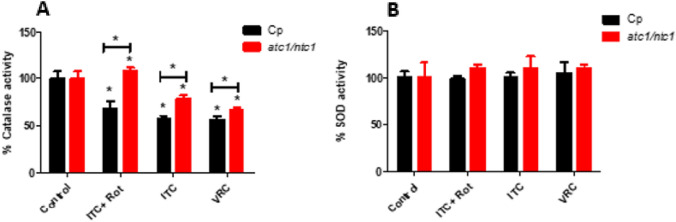
Effect of rotenone on the ITC-induced changes in enzymatic activity shown by the antioxidant enzymes catalase (**A**) and superoxide dismutase (SOD) (**B**) in *C. parapsilosis* Cp and *atc1Δ*/*ntc1Δ* strains. Exponential cultures grown in YPD were subjected to the indicated ITC treatment for 1 h with or without preincubation with rotenone. A sample treated with Voriconazole (VRC) was also included. The data shown are the mean ± SD of three independent experiments. *P < 0.05, represents statistically significant differences with respect to an untreated control according to Mann–Whitney *U* test or between the samples indicated with the brackets

The detection of internal oxidative stress in yeasts is rapidly replied by mounting a general antioxidant response, which involves the activation of enzymatic activities with a detoxifying role (Nikolau et al. 2009). Here, we measured the activity of catalase and superoxide dismutase (SOD) after ITC addition. As shown in Fig. 4A, catalase activity underwent a clear decrease after exposure to ITC compared with untreated controls in parental cells, a diminution that was lower in the *atc1Δ/ntc1Δ* mutant. Notably, pretreatment with rotenone during 1 h before ITC supply induced a partial recovery of catalase in Cp cells, while in trehalase-deficient cells this antioxidant activity was even slightly higher respect to control samples (Fig. 4A). Similar results were obtained when VRC was introduced instead of ITC (Fig. 4A). In turn, the changes recorded in SOD activity, with or without rotenone exposure, were virtually imperceptible, although the trend of a somewhat larger increase in homozygous *atc1Δ/ntc1Δ* cells was maintained (Fig. 4B), suggesting that this enzyme may play a minor role in counteracting the ITC-induced inner oxidative stress in *C. parapsilosis*.

## Discussion

The incorporation during recent decades of new triazolic compounds (Voriconazole, Posaconazole or Ravuconazole) together with the availability of classical azoles (Imidazoles, Fluconazole or Itraconazole) has augmented the limited array of antifungal molecules that can be applied in clinical practice against disseminated infections caused by highly prevalent fungi, even though the antifungal action of azoles is essentially fungistatic rather than fungicide (Campoy and Adrio 2017). More importantly, in addition to the search for new, powerful and safer antimycotic substances, an alternative experimental approach has focused on detailed studies of the mechanism(s) of action evolved by the main clinical antifungals, about which our knowledge is still incomplete (Denning and Bromeley 2015). Thus, the generation of an internal oxidative stress through the formation of ROS is a universal action mechanism of the polyene Amphotericin B (González-Párraga et al. 2011; Mesa-Arango et al. 2014) apart from its well-established binding to membrane ergosterol and subsequent pore formation (Brajtburg et al. 1990; Kontoyiannis and Lewis 2002). However, in the case of azoles preliminary evidence points to the involvement of ROS formation as pathogenic factor besides the main inhibition of ergosterol biosynthesis in *Aspergillus* and *Candida* (Sangalli Little et al. 2011, Belenky et al. 2013, Shekova et al. 2017). Conversely, Micafungin (an echinocandin) is able to stimulate the immune response and the activation of human macrophages, probably through the exposure of β-glucans on the cell wall surface, without the participation of mitochondrial activity (Guirao-Abad et al. 2018).

Regarding the antifungal treatments directed at the pathogenic yeast *C. parapsilosis*, no standard protocol has been defined yet, and therapies vary as a function of the geographic areas and the outbreaks examined (Toth et al. 2019). As a guide, *C. parapsilosis* shows moderate resistance to echinocandins and some clinical isolates are refractory to azoles. We have reported a certain toxic effect of Itraconazole (ITC) on *C. parapsilosis*, which was increased by disruption of the two genes encoding trehalase activities (Sánchez-Fresneda et al. 2022). To verify the putative role of mitochondrial activity as a recently unveiled mechanism involved in the antifungal action of ITC on *C. parapsilosis*, we blocked the first step of the electron transport chain through the specific inhibition of Complex I with subtoxic concentrations of rotenone. According to our results, this approach induced a marked protective effect on cell viability (Fig. 2A and B) and, simultaneously, an important reduction in intracellular ROS production (Fig. 3), although the measurements of antioxidant enzymatic activities (catalase and SOD) were less conclusive (Fig. 4). Alternatively, preincubation with the protonophore DNP, which is an inhibitor of the oxidative phosphorylation, also counteracted the toxic effect of ITC, but to a minor extent (Fig. 2C). Taken together, our data strongly support a main role of mitochondrial activity in the toxic action caused by the addition of ITC on *C. parapsilosis*. It should be noted that, at present, this proposal cannot be extended to other azoles currently used in antifungal therapy. In fact, our previous analysis with the exposure to rotenone plus Fluconazole provided no successful results (Zhang et al. 2015; Sánchez-Fresneda et al. 2022), although DNP is able to increase the resistance to Fluconazole (Kontoyannis 2000), whereas indirect evidence from the use of Voriconazole, obtained by determination of both cell viability and antioxidant enzymatic activities seems more supportive with the involvement of mitochondrial activity (Figs. 1 and 4).

On the other hand, our contribution also reinforces the accomplishment of new research efforts on the central nutritional and physiological pathways in pathogenic fungi as promising therapeutic targets, because some of them are directly involved in the initial infection and further colonization of specific niches inside the host (Prieto et al. 2014; Correia et al. 2019; Wijnants et al. 2020; 2022). Thus, in *C. albicans*, it has long been known that mutants unable to develop true hypha are avirulent, suggesting that formation of mycelial structures is required for a productive tissue invasion (Lo et al. 1997). Furthermore, in the glycolytic pathway, sugar phosphorylation appears to control the virulence in mice (Wijnants et al. 2020), whereas a regulated depletion of fructose-1,6-bisphosphatase, a key enzyme also involved in gluconeogenesis, hampers the growth and capacity of infection in another mouse model (Rodeki et al. 2006). It seems reasonable, therefore, to propose that manipulation of bottleneck enzymes as well as the design of potent and safe inhibitors against crucial enzymatic steps of the central pathways may be regarded as a valuable way toward attaining efficient antifungals for use in clinical practice.

## Data Availability

The completed set of data or minor details presented in this study are available on request from the corresponding author. A publicly accessible repository has not been created.

## Abbreviations

AmB: Amphotericin B
ITC: Itraconazole
VRC: Voriconazole
DNP: 2,4-Dinitrophenol
MICs: Minimum Inhibitory Concentration
PBS: Phosphate Buffer Saline
ROS: Reactive Oxygen Species
SOD: Superoxide Dismutase
